# Oral Mass of a Fetus Incidentally Found during Second Trimester: Ultrasound Survey and Postnatal Prognosis of the Baby

**DOI:** 10.1155/2018/6590710

**Published:** 2018-02-06

**Authors:** Huseyin Durukan, Savas Gundogan, Murside Çevikoğlu Kıllı, Talat Umut Kutlu Dilek

**Affiliations:** ^1^Faculty of Medicine, Obstetrics and Gynecology, Mersin University, Mersin, Turkey; ^2^Faculty of Medicine, Obstetrics and Gynecology, Acibadem University, Istanbul, Turkey

## Abstract

Ultrasound (USG) and magnetic resonance imaging (MRI) can be used to detect and evaluate the face and neck tumors during the in-utero period. We reported and discussed an oral mass which was diagnosed incidentally at mid-trimester exam and managed successfully.

## 1. Introduction

The differential diagnoses of head and neck masses in the fetus include teratoma, lymphangioma, congenital goiter, tumors, cysts of thyroid, neuroblastoma, and hematomas [1]. Invasive diagnostic procedures are suggested for the bilateral head and neck masses because of being associated with genetic anomalies [1]. The most frequent head and neck mass is teratoma [2]. We reported and discussed an oral mass which was diagnosed incidentally at mid-trimester exam and repaired successfully in the infancy.

## 2. Case

**Figure 1:**
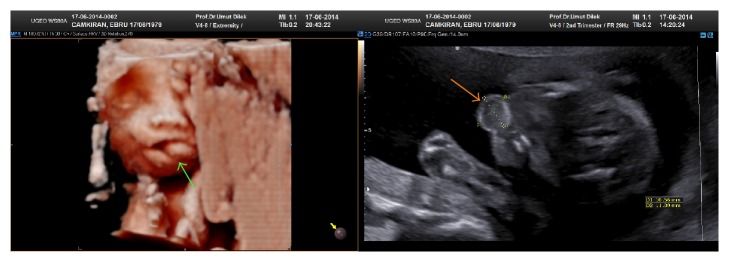
The 3D and 2D views of the mass by USG.

35-year-old, gravida 3 para 0 woman was admitted for the anatomical survey of her fetus at the 21st week of pregnancy. During the US examination, we detected 11 × 10 mm solid mass between tongue and palate. This solid mass was homogeneous and originated from the bottom of the mouth The mass has no abnormal vessel architecture by Doppler US (Figure 1).

**Figure 2:**
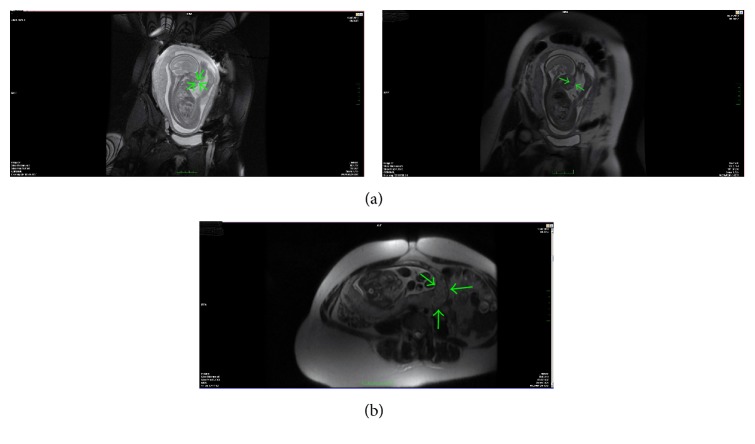
The MRI images of the fetus after first diagnosis of epignathus, green arrows show the mass.

There was no cleft lip. We decided to perform MRI to reveal extension of solid mass and rule out other head and neck anomalies (Figure 2). Lips and hard palate were visualized as intact by both US and MRI. The parents had refused prenatal invasive genetic tests. The couple was informed about the possible risks in the pregnancy. Polyhydramnios did not occur in the remained pregnancy period that could be common. Before cesarean section (C/S) the neonatologists were informed about oral solid mass which might obstruct the airway of the neonate, and the possible need for the EXIT (ex-utero intrapartum treatment) procedure [3].

**Figure 3:**
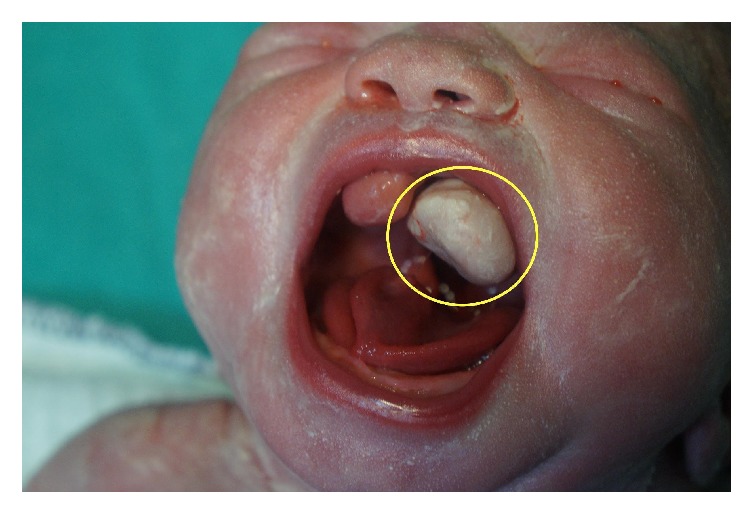
The epignathus, just after delivery, was shown in yellow circle.

The baby was delivered by cesarean section in 37 weeks and 4 days of gestation due to breech presentation. The baby's weight was 2690 gram and had 7 and 9 Apgar scores. In the first neonatal examination of the baby, there was a white, 2 × 2 cm solid mass between the tongue and palate in the mouth (Figure 3). It has smooth surface. There was another pink coloured adjacent soft tissue mass that was evaluated as accessory tongue. Postnatal karyotype analysis was reported as 46 XX. Therefore, considering the relation between epignathus and congenital heart diseases, the postnatal echocardiography was performed and it revealed mild atrial septal defect and mild tricuspid insufficiency [1]. In the postnatal exam, she had low-set ear and flattened-based nose. The postnatal MRI revealed a 26 × 13 × 10 mm soft tissue mass, which has left paramedian location and seemed hyperintense in the T1 planes and isointense in the T2 planes. She underwent surgery in the neonatal period to excise oral solid mass. In the operation, following the excision of oral mass, incomplete cleft palate was surprisingly seen in the soft palate. In the histopathologic examination of the mass, the fat tissue was painted by S100 and surface epithelium with pancreatin [4]. Final histopathologic diagnosis was mature cystic teratoma. She underwent another surgery to excise accessory tongue and repair of incomplete soft palate cleft when she was 14 months old. Postoperative follow-up is normal after second operation.

## 3. Discussion

Teratoma is the most common tumor in infants, especially inside gonads, brain, mediastinum, and sacrococcygeal region. They account only for 6% of tumors in the head and neck region [5]. Epignathus or enigmatic teratomas are masses that originate from palate and growth toward Rathke pouch. Other teratomas arise from tonsils or bottom of the tongue [6]. Oral mass can be the cause of cleft palate by being barrier on the way of embryonic plate migration road [1, 6]. The masses of fetal mouth can be diagnosed in the very early stage due to persistent opened fetal mouth. Polyhydramnios can be a frequent sign and complicates pregnancy when the mass impedes the fetus to swallow the amniotic fluid. Large oral mass could block the airway of the fetus, so following the delivery having an opened airway is critical [7]. Early recognition and identification of the need for postpartum intervention and antenatal management are important. MRI could determine both, expansion of oral mass and obstruction of airway or not. We confirmed a single oral mass which occupies only mouth by both 3D and 2D ultrasound and MRI. 3D ultrasound can give us further information about head and neck masses as successful as MRI. In case of very large tumors, multidisciplinary approach is needed for a safe labor during the delivery [8]. This is a part of OPPS (operation on placental support), which includes the endotracheal intubation after fetal head and shoulder come out of the uterus while the rest of the baby is still inside the uterus and the uterus is kept relaxed by specific agents such as Terbutaline or nitroglycerin [3]. In our case, oral mass did not cause airway obstruction and bleeding. We did not need to perform EXIT procedure because airway of baby was not compromised.

Surgical excision is the management of choice. Following the surgery, prognosis is generally good without recurrence and risk of malignancy. Despite the fact that maxillofacial surgery is complex, both excision and reconstruction may be necessary [9].

This case indicates that prenatal diagnosis and anatomical details of oral mass are possible by both 3D ultrasound and MRI. Also postnatal approach could be determined by imaging techniques prenatally. Because of superimposing or concealing oral masses, the diagnosis of cleft in soft palate can be missed.
